# Management of arthralgias associated with aromatase inhibitor therapy

**DOI:** 10.3747/co.2007.152

**Published:** 2007-12

**Authors:** C. Thorne

**Keywords:** Arthralgia, arthritis, aromatase inhibitors, breast cancer

## Abstract

For the upfront adjuvant therapy of postmenopausal estrogen receptor–positive breast cancer, the third-generation aromatase inhibitors (ais) have shown a more favourable overall risk–benefit profile than has tamoxifen. Benefits of the ais include less frequent gynecologic, cerebrovascular, and thromboembolic adverse events; greater disease-free survival; and lower tumour recurrence. Although approximately 25% of postmenopausal women with early breast cancer report experiencing symptoms of arthralgia with ai therapy, 68-month data from the Arimidex, Tamoxifen, Alone or in Combination trial showed that, compared with tamoxifen, anastrozole treatment was associated with only a modest increase in the incidence of joint symptoms. The events, which were mostly mild-to-moderate in intensity, led to treatment withdrawal in 2% of patients on anastrozole as compared with 1% in the tamoxifen arm. The symptoms and changes correlate with clinical, biochemical, and radiologic findings in symptomatic women. To determine appropriate intervention, it is therefore essential to perform a comprehensive evaluation of musculoskeletal complaints to distinguish natural menopause-related degenerative disease from ai-related effects. The present review explores the advantages of differential diagnosis with an emphasis on history and physical and musculoskeletal examination; laboratory investigations are used to corroborate or rule out clinical impressions. The transient symptoms associated with the ais are manageable with an appropriate combination of lifestyle changes, including exercise and joint protection in conjunction with pharmacologic approaches.

## 1. INTRODUCTION

The benefit of aromatase inhibitors (ais) in the treatment of hormone-dependent early breast cancer is now well established, leading to a recommendation for their use as adjuvant therapy in postmenopausal women 1–4. The underlying rationale for adjuvant treatment of early-stage breast cancer following primary therapy is to prevent or delay recurrence, although the goal is also to increase the time to progression of the tumour and to improve overall survival. Although disease-free survival is approximately 5 times longer in cases of early-stage disease than in cases of metastatic breast cancer 5, patients at early stages of breast cancer who receive adjuvant endocrine therapy still need to understand the safety and toxicity of the treatment.

## 2. MUSCULOSKELETAL COMPLAINTS WITH AIs

Although the ais are associated with fewer thromboembolic events and endometrial abnormalities than is tamoxifen, approximately 25% of postmenopausal women on ais report arthralgia, skeletal, and muscle pain 6.

The multicentre double-blind placebo-controlled ma.17 trial by the National Cancer Institute of Canada Clinical Trials Group compared 5 years of letrozole therapy with 5 years of placebo in 5187 postmenopausal women with breast cancer. The trial revealed marked increases in arthralgia (25% vs. 21%) and myalgia (15% vs. 12%) in patients on letrozole as compared with those on placebo 7.

The Intergroup Exemestane Study, which enrolled 4742 postmenopausal women with hormone receptor–positive or unknown breast cancer, has also shown a link between arthralgia and the ai exemestane (5.4% exemestane vs. 3.6% tamoxifen) 8. The Arimidex, Tamoxifen Alone or in Combination (atac) trial, which involved 9366 postmenopausal women with localized breast cancer following surgery, was one of the largest trials to investigate a third-generation ai as adjuvant therapy for breast cancer. In atac, after 68 months’ median follow-up, the incidence of arthralgia was significantly higher in the anastrozole group than in the tamoxifen group [1100 of 3092 (35.6%) vs. 911 of 3094 (29.4%) patients] 9. In fact, as compared with tamoxifen, anastrozole treatment was associated with only a modest increase in the incidence of arthralgia. Nearly half (46%) of patients with arthralgia syndrome reported exacerbation of a pre-existing joint condition. Symptoms appeared within the first 2 years of ai therapy (anastrozole 68%, tamoxifen 59%), with a peak incidence at 6 months (anastrozole 29%, tamoxifen 20%). However, half of the patients who recovered were symptom-free within 6 months of onset, and in 75% of patients, symptoms resolved within 18 months. Most events were mild to moderate in intensity, and only a small fraction of patients (2% with anastrozole and 1% with tamoxifen) discontinued treatment because of bone symptoms 10.

### 2.1 Menopausal Arthritis and AI-Induced Arthralgia

The prevalence of joint pain in the knees, hands, feet, and other joints increases with age in women, reaching a maximum in the group 50 to 59 years of age, implicating hormonal changes 11,12. The prevalence of osteoarthritis increases around menopause 13, and rheumatoid arthritis has its highest incidence and prevalence in the decade following menopause, perhaps triggered by estrogen deprivation 14.

The physiologic underpinnings of joint pain involve activation, by inflammatory mediators such as prostaglandins and bradykinin, of the nociceptive fibres innervating the articular structures. Activated nociceptors increase sensitivity to pain caused by a stimulus, including a mechanical stimulus. Inflammation leading to expansion of the nociceptor’s receptor field enhances neural transmission and the overall pain perception 15. The neuroprotective, anti-inflammatory 16,17 and anti-nociceptive activities of estrogen are believed to be mediated by estrogen receptors present in the spinal cord and brain 18. Low estrogen levels (resulting from aromatization of androgens) may therefore have a protective effect by directly affecting opioid pain fibres in the central nervous system and preventing inhibition of nociceptive neurons 19. Estrogen depletion during ai therapy of postmenopausal early and advanced breast cancer results in enhanced nociception and higher rates of arthralgia than are seen with tamoxifen 8,20–22.

Unfortunately, coexisting conditions such as degenerative joint disease, fibromyalgia, osteoporosis, and degenerative disc disease contribute to worsening of the arthralgia or bone pain syndrome 23. Also, arthralgia is a known side-effect of some medications, including angiotensin converting-enzyme inhibitors, proton pump inhibitors, quinolones, and tibolones. Postmenopausal status is also an independent predictor of increased musculoskeletal pain, with estrogen deprivation contributing to arthralgia during menopause and aromatase inhibition exacerbating painful symptoms by further estrogen reduction 24.

The complex challenge facing the physician is therefore to accurately distinguish arthralgia associated with ai therapy from bone disease and inflammatory and degenerative arthropathies, and from pain secondary to other diseases. Tables I and II summarize the various features of articular and nonarticular, and inflammatory and non-inflammatory joint pain respectively.

### 2.2 Clinical Evaluation of Musculoskeletal Complaints

**FIGURE 1:**
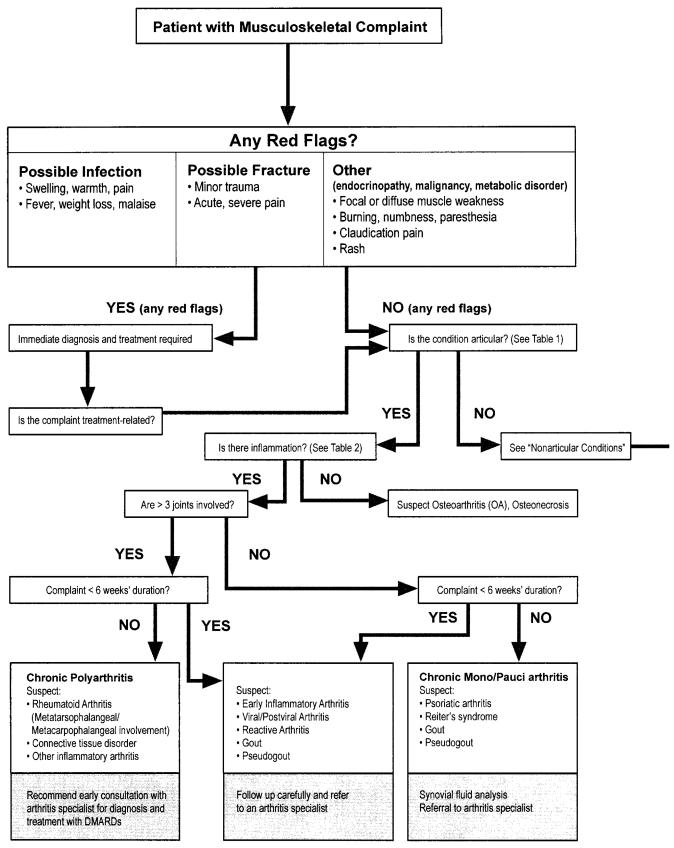

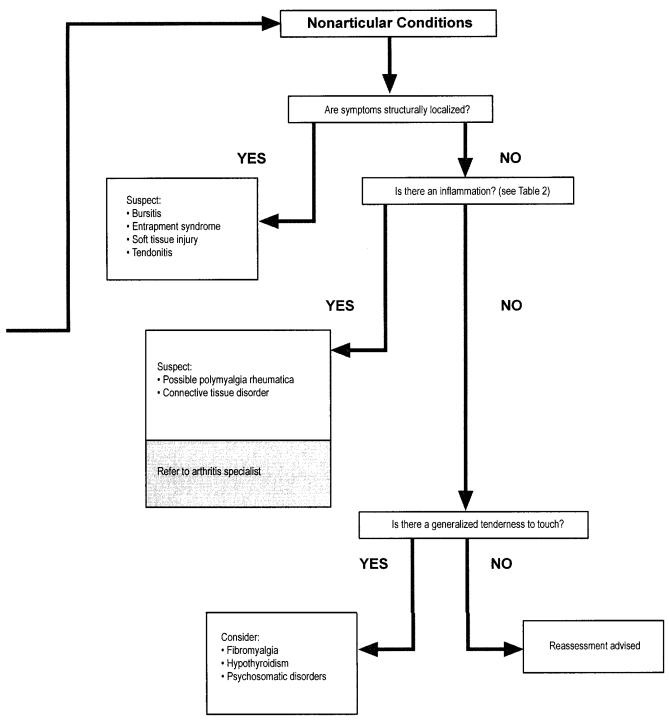
Algorithm for assessment of musculoskeletal complaints (adapted^25^,^28^,^29^).

In the preliminary assessment of musculoskeletal complaints, the emphasis is on patient history and physical examination 26. Patients may describe stiffness, aching, or pain in the hands, arms, knees, feet, pelvic and hip bones, or back, which may be temporally associated with the start of ai therapy. This discomfort is usually symmetrical, and may be associated with mild soft-tissue thickening 27. The physician should rule out conditions that require immediate attention, including a history of significant trauma, a hot and swollen joint, acute severe pain, fever, weight loss, malaise, focal or diffuse muscle weakness, burning, numbness, paresthesia, and claudication 26,28 (Figure 1).

The next step entails determining whether the condition is articular (pauci-articular, involving 3 or fewer joints, or poly-articular, involving more than 3 joints) or non-articular; inflammatory or non-inflammatory; and acute (less than 6 weeks’ duration) or chronic (6 weeks’ duration or longer). Complaints of “joint pain” may originate from the joint itself or from periarticular loci such as adjacent bone, ligaments, tendons, bursae, or soft tissues (Figure 1) 25,28.

#### 2.2.1 Medical History

Appropriate medical history should include investigation of the co-morbidities linked with musculoskeletal complaints: inflammatory bowel disease (ibd), psoriasis, reactive arthritis, hemochromatosis, and hypothyroidism. All medications should be reviewed as completely as possible, including diuretics, antihypertensives, anticonvulsants, cholesterol-lowering agents, and others. Social history should be examined for work, sexuality, living, travel, and familial patterns of connective tissue disease, psoriasis, ibd, and gout. A systems review should also be undertaken to note any other symptoms or infections of the gastrointestinal, genitourinary, ocular, and skin systems 25.

#### 2.2.2 Physical and Musculoskeletal Examination

During the physical examination, the physician should check for the presence of extra-articular features, including nodules, tophi, or skin rashes (psoriasis), or the presence of renal, pulmonary, neurologic, or ocular signs or symptoms. Any joint effusion may be clinically significant and should be observed expectantly, except when septic arthritis, osteomyelitis, fracture, mechanical derangement, or tumour is suspected. Lyme arthritis should be considered in patients with new persistent inflammatory features—including effusions—in regions where Lyme disease is endemic, or in patients with a history of travel to an endemic area. Synovitis is usually indicated by the presence of “stress pain” or an effusion.

Mal-alignment and bony enlargement may suggest advanced osteoarthritis. On the other hand, joint tenderness with full and painless passive range of motion may indicate a peri-arthritis, including bursitis, tendonitis, tenosynovitis, or enthesitis, but no inflammation of the synovial lining of the joint space (Figure 1, Table ) I 25.

#### 2.2.3 Inflammation

The pattern and number of inflamed joints should be observed: acute monoarthritis may represent septic arthritis or crystal arthritis (gout, for instance; Table II). Oligoarthritis or polyarthritis involving the hands and wrists but not the distal interphalangeal joints is suggestive of rheumatoid arthritis. Early symptoms of rheumatoid arthritis include morning stiffness (more than 30 minutes) and three or more swollen joints accompanied by metacarpophalangeal or metatarsophalangeal involvement, which may be simply demonstrated with the “squeeze test” 29.

Psoriasis and osteoarthritis should be suspected if the distal interphalangeal and proximal interphalangeal joints are involved. Gonococcal arthritis, *Rubella,* and acute rheumatic fever may present with a migratory polyarthritis. Spondyloarthropathy such as ankylosing spondylitis, reactive arthritis (formerly Reiter syndrome), psoriatic arthritis, or arthropathy of ibd involve peripheral joints, enthuses, and whole digits (dactylitis).

Myalgias and stiffness of the shoulder and pelvic girdle that occur in patients more than 50 years of age may be suggestive of polymyalgia rheumatica. Infection, metastatic malignancy, or neurologic causes must be ruled out when evaluating new low back pain in this group of patients.

#### 2.2.4 Laboratory Investigation

The laboratory test that is most useful for evaluating patients with inflammatory arthritis and an effusion is synovial fluid analysis (Table II). The erythrocyte sedimentation rate (esr) and C-reactive protein (crp) test are non-specific and may be elevated with other conditions, including malignancy and infection. Rheumatoid factor is also non-specific and increases with age, regardless of the presence or absence of joint disease. Patients with connective tissue diseases, diffuse musculoskeletal symptoms, or drug-induced diseases may have positive antinuclear antibodies.

### 2.3 Investigation of AI-Associated Arthralgia

The pathogenic and anatomic features of ai-induced arthralgia have not been clearly delineated, but the musculoskeletal symptoms and changes have been investigated at the clinical, biochemical, and radiologic levels in symptomatic women. For instance, after an average of 8 weeks’ treatment, the symptoms most commonly reported with ais such as letrozole or exemestane have been severe early morning stiffness and hand–wrist pain. Clinical signs included severely limited mobility in the affected part of the hand or wrist. “Trigger finger” and carpal tunnel syndrome were the most frequently reported clinical signs, but inflammatory disease was ruled out in the absence of elevation in crp or esr, although those tests have not been assessed for sensitivity or specificity in this population. However, ultrasound examination showed fluid in the tendon sheath surrounding the digital flexor tendons. Magnetic resonance imaging (mri) in various patients showed fluid in the tendon sheaths of the digital flexor muscles, intra-articular fluid accumulation in the metacarpal joints, and synovitis of the radiocarpal joint 6. Because of the prevalence of abnormal or unexpected findings in asymptomatic patients, mri of the spine and computed tomography 30 should be reserved for patients targeted for surgical intervention or those in whom tumour or infection is a concern. Laboratory and radiographic studies should be used to corroborate clinical impressions or to rule out causes that should not be missed. Systematic clinical re-evaluation is imperative in the event of uncertain diagnosis.

### 2.4 Management of Arthralgia

Studies reveal that the three most common sites of arthralgia, in descending order, are knees, hands and wrists, and shoulders, with the median severity scores of ai-related joint symptoms being 7 for pain and 6 for stiffness on a scale of 0–10. However, more than half of patients with ai-associated joint symptoms reported relief with medications or supplements. Of those taking an analgesic, 41% took acetaminophen, 59% took a nonsteroidal anti-inflammatory drug (nsaid), and 12% took other medications. The median score for relief achieved with oral medications was 7. In addition, 55% of patients used non-pharmacologic interventions, mainly exercise, to alleviate joint symptoms 31.

Compared with patients receiving tamoxifen, patients receiving anastrozole had only a modest increase in joint symptoms, and most reported symptoms that were mild to moderate in intensity, eliminating the need for treatment withdrawal. Indeed, more than 90% of patients treated for anastrozole-induced joint aches and pains were managed with an nsaid alone or in combination with mild analgesics 10.

**FIGURE 2:**
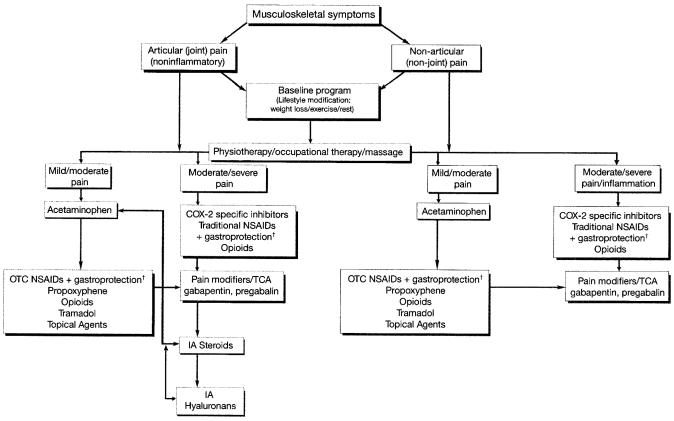
Management of musculoskeletal symptoms in patients with early breast cancer taking an aromatase inhibitor. ^†^ Misoprostol or proton pump inhibitor. cox-2 = cyclooxygenase 2; ia = intra-articular; nsaid = nonsteroidal anti-inflammatory drug; otc = over-the-counter; tca = tricyclic antidepressant.

The most appropriate intervention for pain management in ai-associated arthralgia may be a combination of lifestyle changes, such as introducing weight-bearing exercise, abstaining from smoking, being moderate in alcohol consumption, and taking dietary calcium supplements and vitamin D for bone protection in conjunction with pharmacologic approaches. Figure 2 contains a proposed algorithm for the management of musculoskeletal symptoms in patients taking an ai 32.

#### 2.4.1 Non-pharmacologic Approaches to the Management of Arthralgia

Chronic pain may be relieved either with heat, in the form of warm showers or hot massage, or with acupuncture and acupressure techniques or transcutaneous electrical nerve stimulation 33. However, patients with spinal osteoporosis should avoid deep muscle massage 33. Preliminary results in more than a dozen eligible patients investigated at the Columbia University College of Physicians and Surgeons 34 suggested that acupuncture could be a potentially useful intervention for improvements in index measures of body pain and osteoarthritis.

Pain associated with arthralgia of the knees may also be managed through patellar taping and appropriate footwear with lateral wedged insoles (Plourde P Arthralgia in postmenopausal breast cancer patients on adjuvant endocrine therapy: a risk–benefit analysis. Poster presentation at the Lynn Sage Breast Cancer Symposium; Chicago, IL; October 6–9, 2005). Furthermore, regular stretching, joint mobility exercises and physiotherapy to regain strength, increase flexibility, relieve tension, and reduce fatigue are recommended. In addition, psychotherapy including relaxation training, biofeedback, visual imagery, distraction methods, and psychiatric intervention could effectively alleviate pain-related emotional stress and depression 33, given the increased mood, anxiety, and substance use disorders seen in patients with musculoskeletal disorders, according to a large Canadian community health survey 35.

#### 2.4.2 Pharmacologic Approaches

Table III presents some pharmacologic options for pain relief. Drug therapy for amelioration of pain symptoms includes acetaminophen, nsaids such as naproxen, mild opiates, strong opiates, and glucosamine 23. Combination analgesics (for example, acetaminophen with codeine or oxycodone) may be used to provide additional short-term relief 28. Inhibitors of cox2 may be a good choice when nsaids are contraindicated. As well, topical medications such as capsaicin and methylsalicylate may provide pain relief. Antidepressants may help in the event of comorbid depression associated with chronic pain 36.

Bisphosphonates have been associated with modest adjunctive analgesic benefits, but little clinical evidence is available to support their use in early-stage breast cancer 37,38. By helping to rebuild the microarchitecture of bone, bisphosphonates may offer women with early breast cancer some relief from arthralgia 39. The correlation of ai-associated arthralgia with a low body mass index is still not clearly established, although several possible mechanisms associate the phenomenon with an incidence of microfractures and local activation of inflammatory mediators.

Patients with severe pain that is refractory to simple analgesics or nsaids, and those who present with new joint pain symptoms require a rheumatologic evaluation to rule out inflammatory conditions.

## 3. CONCLUSION

Adverse events such as arthralgia and myalgia are more frequent with adjuvant ai therapy than with tamoxifen. However, they are predictable and may be easily managed with appropriate lifestyle changes and non-pharmacologic interventions, or with pain medications after accurate musculoskeletal evaluation and differential diagnosis. Although a few studies suggest the use of bisphosphonates to manage bone-related side effects 40, current evidence is insufficient to support the use of those agents in preventing skeletal events or improving survival in women with early-stage breast cancer 37. Although the association of estrogen deprivation with osteoarthritis and rheumatoid arthritis in postmenopausal women needs further study, it is essential to understand that the symptoms are usually transient and resolve when the ais are discontinued at the end of therapy.

## Figures and Tables

**TABLE I:** Articular compared with nonarticular disorders 25

Articular pain or tenderness	Nonarticular pain
Joint-specific Exacerbated with passive and active movement Specific referral patterns Frequent swelling: synovial effusion, thickening, and bony enlargement Crepitation may be present Frequent mechanical symptoms—for example, locking, instability Radiographic changes common in chronic and acute conditions except trauma	Originate from periarticular structures (tendons or bursae) Exacerbated with active range of motion Unusual symptoms and pain referral patterns Multiple somatic complaints Type of pain typical of fibromyalgia, hypochondriasis, and pain amplification syndromes Uncommon

**TABLE II:** Inflammatory compared with non-inflammatory joint pain 25

Inflammatory	Non-inflammatory
*Clinical features*	
Joint pain with activity and rest	Joint pain improves with rest and worsens with activity
Morning stiffness >1 h	Morning stiffness <1 h
Inflamed joints can lead to contractures	Contractures unlikely
Crepitus may occur in inflammatory arthritis (felt as “soft”)	Crepitus usually absent (if present, “hard crepitus” bone on bone)
Radiologic changes seen	—
*Optional investigations*	
esr, crp elevated	esr, crp normal
Synovial fluid cloudy	Synovial fluid normal, straw coloured
Synovial fluid WBC > 2000/μL	Synovial fluid WBC < 2000/μL
Synovial fluid neutrophils > 75%	Synovial fluid neutrophils < 75%
Radiologic investigations: MRI, CT if tumour or infection is suspected or if surgery is contemplated	—
*Conditions*	
Rheumatoid arthritis	Osteoarthritis
Psoriatic arthritis	Trauma
Spondyloarthropathies	Glucocorticoid withdrawal
Gout	Hypertrophic osteoarthropathy
Pseudogout	Avascular necrosis
Systemic lupus erythematosus	Pigmented villonodular synovitis
Septic arthritis	Hemochromatosis Acromegaly Charcot’s joint

esr = erythrocyte sedimentation rate; crp = C-reactive protein; wbc = white blood cells; mri = magnetic resonance imaging; ct = computed tomography.

**TABLE III:** Pharmacologic options for amelioration of pain in early breast cancer patients with symptoms of arthralgia

Medication	Dosage
Acetaminophen a	≤daily (consider er)
nsaid a	Naproxen: 500–750 mg daily (analgesia) 1000–1500 mg daily (inflammation)
Cyclooxygenase inhibitors a	Celecoxib: 100–200 mg daily b
Tramadol	≤400 mg daily (consider er)
Narcotics	Codeine versus oxycodone
Pain modifiers	tca: nortriptyline 10–100 mg daily Gabapentin, pregabalin
Intra-articular steroids	Methylprednisolone acetate, triamcinolone suspension: 40 mg/cm^3^

a Minimum 2 weeks at tolerated dose.

b Maximum 400 mg daily.

nsaid = nonsteroidal anti-inflammatory drug; er = extended release; tca = tricyclic antidepressant.
